# Interments in the City and Liberties of Philadelphia, from the 28th of August to the 4th of September

**Published:** 1841-09-11

**Authors:** 


					﻿HEALTH OF THE CITY
Interments in the City and Liberties of Phila-
delphia, from the W>th of Jlugust to the 4.th
of September.
> O	> O
Diseases.	£ Diseases.	S
“re	“re
p	p
Apoplexy,	1	0	Brought forward,32	43
Casualty,	0	1	Inanition,	0	1
Croup,	0	2	Marasmus,	0	7
Congestion of	Mania a Potu, 1	0
Lungs,	0	1	Rupture of Uterus, 1	0
____Brain, 0	1 Softening of Sto-
Cholera Morbus,	0	1	mach,	0	1
Colic, Painters’	1	0	Small pox,	2	4
Consumption of	Spina Bifida,	0	1
the lungs,	12	1	Still-born,	0	7
Convulsions,	0	4 Suicide,	1	0
Diarrhoea,	1	9	Summer	Com-
Dropsy,	2	0	plaint,	0	22
____nf the	Tabes Mesente-
head,	0	5	nca,	0	1
Disease of the	Unknown,	0	1
heart,	1	0|
Dysentery,	1	5	Total,	125—37	88
Debility	0	1	------
Fracture of skull, 0	1 Of the above, there
Fever,	0	1 were under 1 year, 52
----Bilious,	1 0	From 1	to	2___24
___________________________________________Typhus,____________________________________2 0_________________________________2________________________________to______________________________5_____________________________3
___________________________________________Typhoid,	10	5	to	10	5
■ Scarlet,	02	10	to	15	2
Hernia,	10	15 to 20	2
Inflammation of	20 to 30	12
the Brain,	1	3	30	to	40	8
----Lungs,	1	2	40	to	50---10
------------------------------------------ Stomach,---------------------------------1--------------------------------0-------------------------------50-----------------------------to---------------------------60-------------------------0
------------------------------------------Bowels, 2---------------------------------1--------------------------------60 to /0------------------------4
------------------------------------------Peritonaeum,0	2	70	to	80	3
Intemperance,	3	0	80	to	90	0
Carried forward, 32 43 Total,	125
				

